# Suppressing Manganese Dissolution via Exposing Stable {111} Facets for High‐Performance Lithium‐Ion Oxide Cathode

**DOI:** 10.1002/advs.201801908

**Published:** 2019-04-29

**Authors:** Yao Xiao, Xu‐Dong Zhang, Yan‐Fang Zhu, Peng‐Fei Wang, Ya‐Xia Yin, Xinan Yang, Ji‐Lei Shi, Jian Liu, Hongliang Li, Xiao‐Dong Guo, Ben‐He Zhong, Yu‐Guo Guo

**Affiliations:** ^1^ School of Chemical Engineering Sichuan University Chengdu 610065 P. R. China; ^2^ CAS Key Laboratory of Molecular Nanostructure and Nanotechnology CAS Research/Education Center for Excellence in Molecular Sciences Beijing National Laboratory for Molecular Sciences (BNLMS) Institute of Chemistry Chinese Academy of Sciences (CAS) Beijing 100190 P. R. China; ^3^ Institute for Superconducting and Electronic Materials University of Wollongong Wollongong NSW 2522 Australia; ^4^ Beijing National Laboratory for Condensed Matter Physics Institute of Physics Chinese Academy of Sciences (CAS) Beijing 100190 P. R. China; ^5^ Institute of Materials for Energy and Environment Laboratory of New Fiber Materials and Modern Textile Growing Basis for State Key Laboratory College of Materials Science and Engineering Qingdao University Qingdao 266071 P. R. China

**Keywords:** {111} facets, cathode materials, hollow fusiform structures, lithium‐ion batteries, manganese dissolution

## Abstract

Spinel‐type LiMn_2_O_4_ cathode materials commonly suffer from manganese dissolution due to the severe interfacial side reactions especially at elevated temperature. Here, a 3D hollow fusiform LiMn_2_O_4_ cathode material is reported with preferentially exposed stable {111} facets and seamless outer structure, which is clearly confirmed by microfocused ion beam scanning electron microscopy, high‐resolution transmission electron microscopy as well as scanning transmission electron microscopy with atomic resolution. Owing to the optimal geometrical structure design and the preferentially exposed stable {111} facets, the electrode delivers excellent rate capability (107.6 mAh g^−1^ at 10 C), remarkable cycling stability (83.3% capacity retention after 1000 cycles at 1 C), and outstanding high‐temperature performance. Together with the analyses of electrochemical behaviors, in situ X‐ray diffraction at different temperatures, and ex situ X‐ray photoelectron spectra, the underlying working mechanism for suppressing manganese dissolution is clearly articulated. These findings could provide significant guidelines for precisely designing highly stable cathode materials for LIBs.

In order to meet with the ever‐increasing energy demand, it is urgent to effectively utilize intermittent renewable energy sources (wind, solar, wave, and geothermal).^1^, ^2^, ^3^, ^4^, ^5^, ^6^ Lithium‐ion batteries (LIBs) are considered as one of the most efficient energy‐storage technologies and have attracted intensive attention to realize the application in electric vehicles because of high energy/power density, superior service life, and design flexibility.^7^, ^8^, ^9^, ^10^ Spinel‐type LiMn_2_O_4_ cathode materials have been spotlighted owing to high thermal stability, fast 3D Li^+^ diffusion paths, abundance of the raw materials, good safety, and environmental friendliness.^11^, ^12^, ^13^, ^14^, ^15^ However, the further large‐scale application is hampered by poor cycling performance due to the manganese dissolution, especially at elevated temperature.^16^, ^17^, ^18^, ^19^

To achieve high‐performance cathode materials for LIBs, tremendous research works have been devoted to restraining unfavorable interfacial side reactions. Surface modification and cationic substitution have been demonstrated to be efficient strategies to suppress the manganese dissolution from the cathode/electrolyte interface and avoid the deposition of Mn^2+^ on the anode.^20^, ^21^, ^22^, ^23^ Nevertheless, the coating layers are usually not uniform or continuous, resulting in uncoated area of the cathode material still suffering from hydrofluoric acid attack. Meanwhile, the coating layer and cathode material may separate during Li^+^ intercalation/deintercalation process due to the essential dissimilarity of their crystal structures. Besides, substitution of manganese by Al^3+^ and Ti^4+^ would sacrifice specific capacity because of electrochemically inactive of doping cations. Some pioneers have confirmed that the interfacial side reactions at the cathode/electrolyte interface are largely dependent on the lattice orientation based on the arrangement of atoms. Among the predominant facets, the {111} facts display more positive to electrochemical properties owing to the more dense crystal lattice of manganese atoms and less interaction with electrolyte. More importantly, the solid electrolyte interphase (SEI) formed on the {111} facets is thinner and smoother than those of the {100} and {110} facets, which can alleviate manganese dissolution to enhance the cycling stability.^24^, ^25^, ^26^ Meanwhile, previous studies have demonstrated that the reasonable geometrical structure design, such as double‐shelled hollow microsphere, porous sphere, porous nanosheet, nanofiber, nanotube, core–shell, and yolk‐structured microsphere, is significant to improve rate performance of cathode material.^27^, ^28^, ^29^, ^30^, ^31^, ^32^, ^33^ Therefore, an optimal bifunctional design strategy combining the virtues of geometrical structure modulation and preferentially exposed stable {111} facets could realize high‐performance cathode materials for LIBs.

In this case, we precisely design a 3D hollow fusiform LiMn_2_O_4_ cathode material with the preferentially exposed stable {111} facets and the seamless outer structure. Benefiting from the advantages of the unique structure, which is clearly verified by focused ion beam scanning electron microscopy (FIB‐SEM), high‐resolution transmission electron microscopy (HR‐TEM) with fast fourier transform (FFT), and scanning transmission electron microscopy (STEM), the cathode displays excellent electrochemical performance at different temperature. Based on the results of electrochemical behaviors, in situ X‐ray diffraction (XRD) at different temperatures, ex situ x‐ray photoelectron spectra (XPS), and inductively coupled plasma mass spectrometry (ICP‐MS) after cycles, the phenomenon of suppressing manganese dissolution is obvious. The superior cathode material could be considered as a potential candidate for further large‐scale application in the field of energy storage.

A 3D hollow fusiform LiMn_2_O_4_ cathode material with the preferentially exposed stable {111} facets and the seamless outer structure (hereafter denoted as LMO‐HF) was prepared via a facile chemical‐templating route using MnCO_3_ as self‐template. As control group, LiMn_2_O_4_ cathode material was synthesized through a conventional solid state reaction (hereafter denoted as LMO‐CS). As shown in **Figure** **1**a and Figure S1 (Supporting Information), XRD patterns of LMO‐HF and LMO‐CS cathode materials are ascribed to the cubic spinel structure with *Fd‐*3*m* space group. All sharp peaks suggest that the samples are highly crystalline without undesirable impurities.^14^, ^24^ Meanwhile, XRD technique could be used as a strongly supportive tool in determining preferentially exposed facets. It is interesting to note that the intensity of the (111) peak concerning LMO‐HF cathode material is much stronger than that of the (311) peak. According to the calculated result from XRD patterns, the *I*_(111)_/*I*_(311)_ ratio value of LMO‐HF cathode material is 1.76 times higher than that of LMO‐CS cathode material, which may result from the preferentially exposed {111} facets of LMO‐HF cathode material.^34^ In addition, as displayed in Figure 1b,c and Figure S2 (Supporting Information), the crystal structure of LiMn_2_O_4_ cathode material viewed along different crystallographic directions shows that lithium and manganese atoms occupy the tetrahedral Wyckoff 8a sites and the octahedral 16d sites, respectively. The oxygen atoms are located in the 32e sites with a cubic close‐packed manner.^12^, ^17^ Besides, in order to directly record the formation process of LMO‐HF cathode material, in situ XRD at different temperatures of precursors were executed and detailed analyses are presented in Figure 1d,e and Figure S3 (Supporting Information). It is worth noting that the intensity of the (111) peak is much stronger than that of the (311) peak and the *I*_(111)_/*I*_(311)_ ratio values are about 1.88 times higher than that of LMO‐CS cathode material during the heat preservation and cooling process. Furthermore, the high *I*_(111)_/*I*_(311)_ ratio values could be obtained from the in situ XRD at different temperatures of LMO‐HF cathode material during the whole heat and cooling process (Figures S4−S5, Supporting Information), which demonstrates the preferentially exposed stable {111} facets. In addition, the morphological and structural features of fusiform MnCO_3_ templates were investigated by scanning electron microscope (SEM), transmission electron microscopy (TEM), XRD, and size distribution (Figure 1f,g and Figure S6−S8, Supporting Information). After lithiation and calcination at high temperature, the obtained products were characterized by TEM at different magnifications (Figure 1h,i) and present hollow fusiform structure with average diameters around 382.8 nm and lengths of ≈887.1 nm (Figure S9, Supporting Information). As a comparison, SEM images of LMO‐CS material at different magnifications are displayed in Figure S10 (Supporting Information). Meanwhile, the decomposition process of LMO‐HF cathode material is verified by thermogravimetric (Figure S11, Supporting Information). The mechanism concerning the formation of the unique hollow fusiform structure is originated from the release of CO_2_ and the Kirkendall effect, which can be briefly expressed by the following equation: 4LiOH + 8MnCO_3_ + 3O_2_ = 4LiMn_2_O_4_ + 2H_2_O + 8CO_2_.^33^, ^35^ Besides, the chemical compositions and valence state were analyzed by XPS (Figure S12, Supporting Information) and the uniform distributions of Mn and O elements were confirmed by energy dispersive spectroscopy (EDS) mapping (Figure 1j,k). Meanwhile, the ratios of Li and Mn elements were further investigated by ICP‐MS and the results of LMO‐HF and LMO‐CS materials are consistent with the design principle (Table S1, Supporting Information). As shown in Figure 1l, the crystal structure of LMO‐HF material was characterized by the combined analyses of HR‐TEM image with FFT pattern and the interplanar spacing of clear lattice fringe (≈ 4.7 Å) can be assigned to the {111} facets.

**Figure 1:**
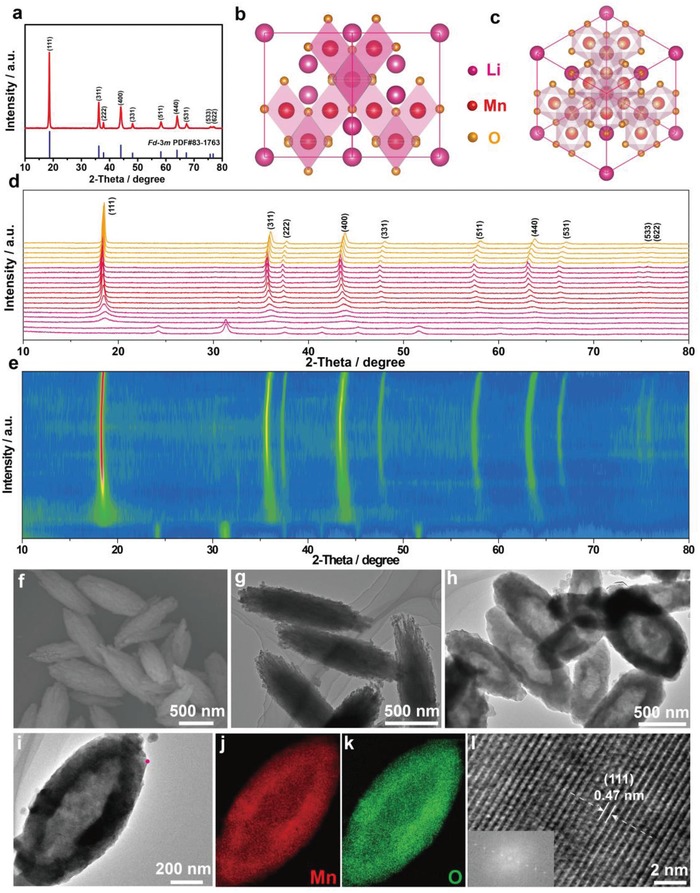
Structure of LMO‐HF material and MnCO_3_ precursor. a) Powder XRD pattern of LMO‐HF material. b,c) *Fd‐*3*m* crystal structure viewed along the [110] and [111] crystallographic directions. d,e) In situ XRD patterns at different temperatures of precursors concerning LMO‐HF cathode material and intensity contour maps concerning the evolution of the main characteristic diffraction peaks. f,g) SEM and TEM images of MnCO_3_ precursor. h,i) TEM images of LMO‐HF material at different magnifications (red dot stands for the position of HR‐TEM image). j,k) EDS mapping. l) HR‐TEM image with FFT pattern as inset.

In order to further confirm the preferentially exposed {111} facets of LMO‐HF cathode material, detailed surface structure analyses of HR‐TEM images with FFT patterns at different sites on randomly selected particle are executed. Obviously, as shown in **Figure** **2** and Figure S13 (Supporting Information), all the HR‐TEM images with corresponding filtered HR‐TEM images of the different areas show clear lattice fringes and all the interplanar spacings are about 0.47 nm, indicating that the exposed planes are {111} facets. Meanwhile, such characteristic fringes can be continually observed in the investigation of as‐prepared LMO‐HF cathode material. Moreover, the clear characteristic spots of FFT patterns further demonstrate cubic spinel structure and preferentially exposed {111} facets (Figure S14, Supporting Information).^24^, ^29^ To further study the surface and interior structure, cross‐sectional FIB‐SEM images from DualBeam instruments at different sites from various angles are displayed in **Figure** **3**a−f. Some octahedral features of primary particles occur as well when we observe the surface of LMO‐HF cathode material carefully. The nanosized subunits with the size of about 50 nm and interior hollow fusiform structure are beneficial for improving rate performance. Meanwhile, the inherent seamless outer structure could minimize the manganese dissolution and interfacial side reactions between cathodes and organic electrolytes by limiting the interfacial area, thereby enhancing structural stability .^36^ As plotted in Figure 3g−i, Figure S15−16, and Video S1 (Supporting Information), 3D reconstructed cross‐sectional video and images at different state are executed via Auto Slice and View with Imaris softwares to provide a better representation of all the information available from 3D hollow fusiform structure.

**Figure 2:**
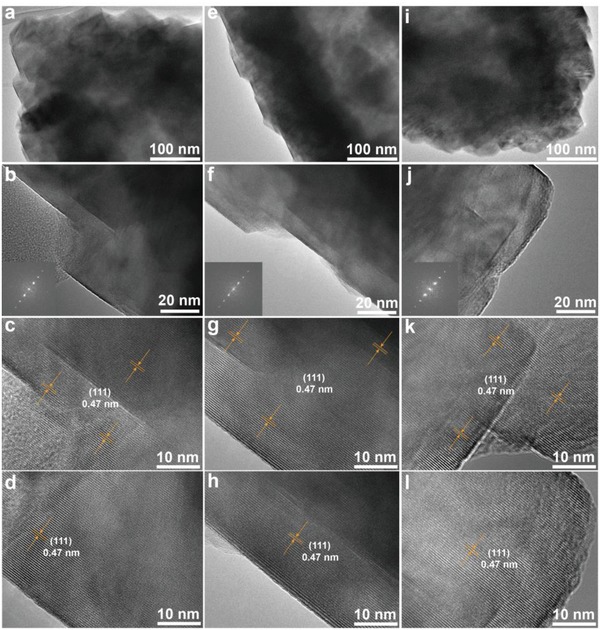
Surface structure of LMO‐HF material. a,b) TEM and HR‐TEM images with FFT pattern as inset. c,d) Enlarged HR‐TEM images at different sites. e,f) TEM and HR‐TEM images with FFT pattern as inset. g,h) Enlarged HR‐TEM images at different sites. i,j) TEM and HR‐TEM images with FFT pattern as inset. k,l) Enlarged image HR‐TEM images at different sites.

**Figure 3:**
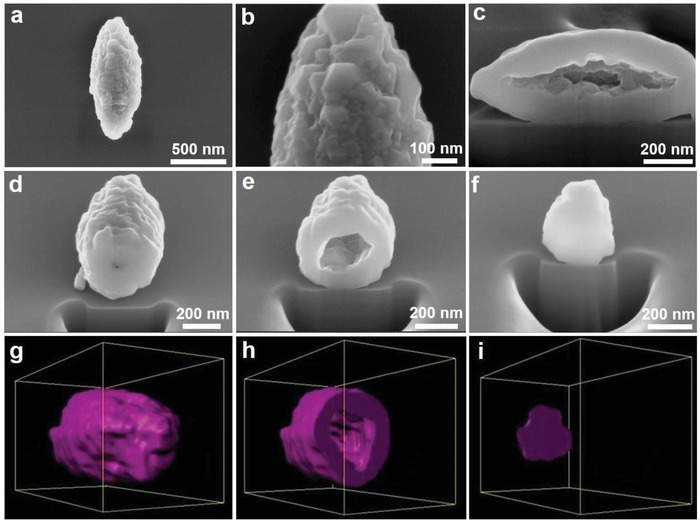
Structure of LMO‐HF material. a−f) Cross‐sectional FIB‐SEM images at different sites viewed from different angles and g−i) corresponding 3D reconstructed cross‐sectional images at different state.

High‐angle annular dark field (HAADF) and annular bright field (ABF)‐STEM were performed by advanced spherical aberration‐corrected electron microscopy to grasp more insights into the local crystal structure with an atomic scale.^37^ The bright‐dot contrast in ABF‐STEM images and the dark‐dot contrasts in HAADF‐STEM images (**Figure** **4**a,b) reveal the transition metal (Mn) atom column positions viewed along the [110] crystallographic direction.^16^, ^18^ As shown in Figure 4c,d and Figure S17−S19 (Supporting Information), the high resolution STEM images, FFT pattern, and line profile reveal the typical spinel atomic arrangements without structure distortion and the two different Mn columns are assigned to Mn1 and Mn2, respectively, which are highly consistent with the atomic models and previously reported results.^12^, ^17^ Based on the analyses of XRD, HR‐TEM, FIB‐SEM, and STEM, a 3D hollow fusiform LiMn_2_O_4_ cathode material with the preferentially exposed {111} facets and the seamless outer structure has been successfully fabricated.

**Figure 4:**
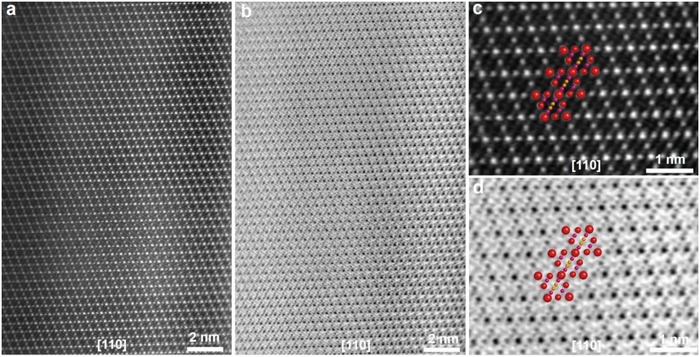
Atomic structure of LMO‐HF material. a,b) HAADF and ABF‐STEM images viewed along the [110] crystallographic direction and c,d) corresponding enlarged HAADF and ABF‐STEM images.

The electrochemical intercalation/deintercalation behaviors of LMO‐HF and LMO‐CS electrodes were tested in Li half cells. As displayed in **Figure** **5**a and Figure S20a (Supporting Information), the LMO‐HF electrode delivers a high discharge specific capacity of 127.9 mAh g^−1^ and excellent specific energy density of 518.4 Wh kg^−1^ at 0.5 C. The typical galvanostatic charge/discharge profiles obviously exhibit two voltage plateaus, which are ascribed to the reactions of λ‐MnO_2_/Li_0.5_Mn_2_O_4_ (4.1 V vs Li/Li^+^) and Li_0.5_Mn_2_O_4_/LiMn_2_O_4_ (3.9 V vs Li/Li^+^), respectively.^38^ Meanwhile, the LMO‐HF electrode was tested by cyclic voltammograms (CV) at 0.1 mV s^−1^ and two pairs of redox peaks are located at 4.05/3.95 and 4.17/4.08 V, respectively, which are consistent with the previously mentioned two voltage plateaus (Figure 5b). To further evaluate the rate capability, the LMO‐HF and LMO‐CS electrodes were investigated at different rate ranging from 1 to 10 C (Figure 5c,d and Figure S20b, Supporting Information). The LMO‐HF electrode could exhibit stable specific capacity with energy density at each current density and still display 107.6 mAh g^−1^ and 428.1 Wh kg^−1^ even at 10 C, whereas the specific capacity of LMO‐CS electrode is merely 76.4 mAh g^−1^ (Figure S21a,b, Supporting Information). Meanwhile, the discharge specific capacity retentions at different rate of LMO‐HF and LMO‐CS electrodes are provided in Figure S21c,d (Supporting Information). Besides, as shown in Figure 5e,f, Figure S22−23, and Table S2−S3 (Supporting Information), the kinetic properties of LMO‐HF and LMO‐CS electrodes were calculated from the quantitative analysis of CV at various sweeping rates at different oxidation and reduction peaks. The LMO‐HF electrode shows higher Li^+^ apparent diffusion coefficient (2.3–4.9 × 10^−10^ cm^2^ s^−1^) and smaller polarization than that of LMO‐CS electrode. Meanwhile, galvanostatic intermittent titration technique (GITT) measurements were used for further investigating the excellent kinetic properties of LMO‐HF electrode, which are also clearly verified by very low average voltage polarization (14.2 mV) and ohmic polarization (8.65 mV) in the first charge process (Figure 5g,h and Figure S24a, Supporting Information). In addition to the rate performance, it significantly displays long‐term cycling stability performance at different temperature. When the temperature is 25 °C, as plotted in the Figure 5i, Figure S24b, and Figure S25 (Supporting Information), the LMO‐HF electrode exhibits a remarkable capacity retention of 83.3% after 1000 cycles with a high coulombic efficiency, stable midpoint voltage, and excellent energy efficiency at 1 C. However, LMO‐CS electrode displays very fast capacity decay (Figure S26, Supporting Information). In addition, as we all know, interfacial side reactions become more serious at 60 °C because of the Jahn–Teller distortion caused by the valence electron configuration of t_2g_^3^e_g_^1^ concerning Mn^3+^ in the octahedral crystal field, leading to poor cyclability. Surprisingly, the LMO‐HF electrode could maintain superior cycle stability at elevated temperatures (71.1% capacity retention after 500 cycles at 0.5 C), which lies at the competitive level (Figure 5j). On one hand, the preferentially exposed stable {111} facets and seamless outer structure could provide dense crystal lattice of manganese atoms, less interaction with electrolyte, and a stable SEI to suppress manganese dissolution, thus enhancing the cyclability especially at elevated temperature. On the other hand, the nanosized subunits of LMO‐HF cathode material could facilitate the Li^+^ intercalation/deintercalation through drastically shortened diffusion paths. Meanwhile, interior hollow fusiform structure could buffer structural strain and volume change at high charge/discharge current.

**Figure 5:**
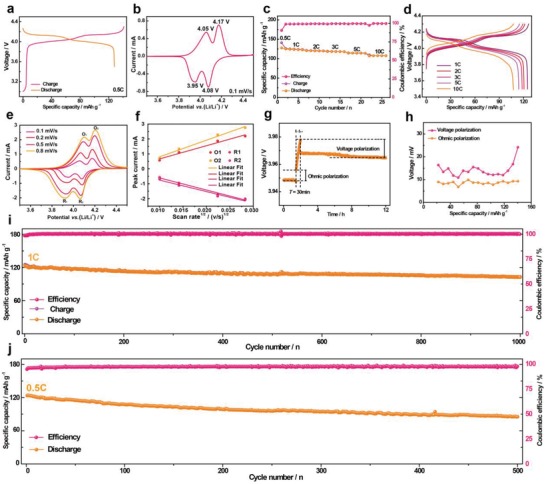
Electrochemical performance of LMO‐HF electrode at different temperature. a) Galvanostatic charge/discharge curves versus specific capacity in the first cycle with a current density of 0.5 C at 25 °C. b) Cyclic voltammogram with a scan rate of 0.1 mV s^−1^ at 25 °C. c,d) Rate performance and corresponding galvanostatic charge/discharge curves versus specific capacity at various rates at 25 °C. e,f) Cyclic voltammograms at different scan rates and the plotting of peak current versus square root of the scan rate at different oxidation and reduction peaks at 25 °C. g,h) A single titration during GITT measurement and voltage with ohmic polarization in the first cycle during the whole charge process at 25 °C. i) Cycling performance during 1000 cycles with a current density of 1 C at 25 °C. j) Cycling performance during 500 cycles with a current density of 0.5 C at 60 °C.

When element manganese dissolves in the electrolyte, they will migrate to the Li metal anode driven by the concentration gradient and/or electric field force. To study the inherent working mechanisms for stabilizing LMO‐HF cathode material, ex situ XPS test was conducted to investigate the elements on the surface of the Li metal anode after 1000 cycles with a current density of 1 C at 25 °C and 500 cycles with a current density of 0.5 C at 60 °C, respectively. Trace element manganese has been detected (Figures S27−S28, Supporting Information) and detailed manganese dissolution amount was tested by ICP‐MS (Table S4, Supporting Information). Manganese dissolution amount of LMO‐HF electrode lies at relatively low level. Meanwhile, as shown in Figure S29 (Supporting Information), it presents the observation of the electrolyte after storage with LMO‐HF electrode at 60 °C for 24 h and no obvious color change can be observed. From these above results, it can be rationally deduced that 3D hollow fusiform structure with stable {111} facets and the seamless outer structure design strategy efficiently suppress the manganese dissolution and avoid the deposition of Mn^2+^ to a certain extent, resulting in excellent performance of cathode materials for LIBs.

In summary, a 3D hollow fusiform LiMn_2_O_4_ cathode material with the preferentially exposed stable {111} facets and seamless outer structure is successfully fabricated via reasonable geometrical structure modulation strategy. On one hand, the special structure not only efficiently facilitates Li^+^ transport to improve the rate performance due to the nanosized subunits, but also contributes to buffering the structural strain and volume change during Li^+^ insertion/extraction. On the other hand, the seamless outer structure could minimize interfacial side reactions between the cathodes and organic electrolytes. Moreover, the preferentially exposed stable {111} facets could provide dense crystal lattice of manganese atoms, less interaction with electrolyte, and a stable SEI to suppress manganese dissolution, thus enhancing the cyclability especially at elevated temperature. As a result, the electrode displays a high discharge specific capacity of 127.9 mAh g^−1^ with superior specific energy density up to 518.4 Wh kg^−1^ at 0.5 C, an excellent rate performance of 107.6 mAh g^−1^ at 10 C, a remarkable capacity retention of 83.3% after 1000 cycles at 1 C, and outstanding high‐temperature performance. This study could offer new insights into the regulation of the physical and chemical properties in spinel oxide cathode materials.

## Experimental Section

*Materials Syntheses*: LMO‐HF cathode material was prepared via a facile chemical‐templating route using fusiform MnCO_3_ as self‐template. More specifically, 2 g KMnO_4_ and 2 g L‐histidine powders were dissolved in 40 mL deionized water, respectively and the mixed solutions were prepared by adding KMnO_4_ solution dropwise into L‐histidine solution under vigorous stirring. The mixed solutions were transferred to a 100 mL Teflon‐lined autoclave and then reacted at 180 ˚C for 12 h. The obtained MnCO_3_ precursors were washed by water and ethanol for several times and then dried in a vacuum oven at 80 °C for 24 h. Finally, a stoichiometric amount of manganese carbonates and lithium hydroxide was dispersed in 20 mL ethanol via ultrasonic and mechanical stirring method, after which the mixture was heated to 80 °C with the continued stirring until excessive ethanol completely evaporated. The powders were calcined at 750 °C for 12 h, then slowly cooled to room temperature to obtain the final product. As control group, LiMn_2_O_4_ cathode material was also synthesized through a conventional solid state reaction.

*Materials Characterizations*: X‐ray diffraction (XRD) pattern was investigated by Bruker D8 Advance Diffractometer with Cu Kα radiation source (λ_1_ = 1.54056 Å, λ_2_ = 1.54439 Å) between 10° and 80°. The precise elementary composition was determined by inductively coupled plasma mass spectrometry (ICP‐MS). Scanning electron microscopy was conducted on a field‐emission microscope (SU‐8020, Hitachi Limited Corporation, Japan) to determine the morphology. High‐resolution transmission electron microscopy images and energy dispersive spectroscopy mapping were examined by TEM (JEM 2100F, JEOL Limited Corporation, Japan) under an acceleration voltage of 200 kV. X‐ray photoelectron spectra were acquired from ESCALab 250Xi (Thermo Scientific) spectrometer equipped with an Al Kα achromatic X‐ray source. High‐angle annular dark field and annular bright field images were executed on JEOL ARM200F (JEOL, Tokyo, Japan) STEM equipped with two CEOS (CEOS, Heidelberg, Germany) probe aberration correctors at 200 kV.

*Electrochemical Measurements*: Electrochemical tests of cathodes were carried out in CR2032 coin‐type half‐cells with lithium metal as counter electrodes, which were assembled in an argon‐filled glove box with water and oxygen contents below 0.1 ppm. Cathode slurry was fabricated by dispersing the as‐prepared cathode material, super P carbon, and polyvinylidene fluoride (PVDF, binder) in *N*‐methylpyrrolidin (NMP) at a weight ratio of 80:10:10. The mixed slurry was spread on an aluminum foil current collector and dried at 80 °C for 12 h in a vacuum oven. The electrode was then punched into disk with an area of 0.785 cm^2^ and the loading mass of active material was about 2.5 mg cm^−2^. The electrolyte was traditional carbonate electrolyte of 1M LiPF_6_ in ethylene carbonate (EC)/dimethyl carbonate (DMC)/diethyl carbonate (DEC) (1:1:1 in volume ratio) and a porous polypropylene film (Celgard 2400) was used to separate the cathode and anode. Galvanostatic tests were performed on Land BT2000 battery test system at different rates (1 C = 147 mAh g^−1^) over a voltage range of 3.5−4.3 V (vs Li^+^/Li) and the cells were charged/discharged at the same current density.

## Conflict of Interest

The authors declare no conflict of interest.

## Supporting information

Supplementary

Supplementary
