# Supplementary material for: Suppressing Manganese Dissolution via Exposing Stable {111} Facets for High‐Performance Lithium‐Ion Oxide Cathode
Source: Adv Sci (Weinh). 2019 Apr 29;6(13):1801908. doi: 10.1002/advs.201801908 (PMC6662411; doi:10.1002/advs.201801908)
Supplement: Supplementary file 1 — Supplementary [file ADVS-6-1801908-s002.pdf]

Copyright WILEY-VCH Verlag GmbH & Co. KGaA, 69469 Weinheim, Germany, 2019.

ADVANCED SCIENCE
Open Access

Supporting Information

for *Adv. Sci.,* DOI: 10.1002/advs.201801908

Suppressing Manganese Dissolution via Exposing Stable {111} Facets for High-Performance Lithium-Ion Oxide Cathode

Yao Xiao, Xu-Dong Zhang, Yan-Fang Zhu, Peng-Fei Wang, Ya-Xia Yin, Xinan Yang, Ji-Lei Shi, Jian Liu, Hongliang Li, Xiao-Dong Guo, Ben-He Zhong, and Yu-Guo Guo**

Supporting information for

Suppressing Manganese Dissolution via Exposing Stable {111} Facets for High-Performance Lithium-Ion Oxide Cathode

Yao Xiao, Xu-Dong Zhang, Yan-Fang Zhu, Peng-Fei Wang, Ya-Xia Yin, Xinan Yang, Ji-Lei Shi, Jian Liu, Hongliang Li, Xiao-Dong Guo*, Ben-He Zhong, Yu-Guo Guo*

Y. Xiao, Y.-F. Zhu, Dr. X.-D. Guo, Prof. B.-H. Zhong
School of Chemical Engineering, Sichuan University, Chengdu, 610065, P.R. China
E-mail: xiaodong2009@163.com

Y. Xiao, Dr. X.-D. Zhang, Dr. P.-F. Wang, Dr. Y.-X. Yin, Dr. J. -L. Shi, Dr. J. Liu, Prof. Y.-G. Guo
CAS Key Laboratory of Molecular Nanostructure and Nanotechnology, CAS Research/Education Center for Excellence in Molecular Sciences, Beijing National Laboratory for Molecular Sciences (BNLMS), Institute of Chemistry, Chinese Academy of Sciences (CAS), Beijing 100190, P.R. China
E-mail: ygguo@iccas.ac.cn

Prof. H. Li
Institute of Materials for Energy and Environment, Laboratory of New Fiber Materials and Modern Textile, Growing Basis for State Key Laboratory, College of Materials Science and Engineering, Qingdao University, Qingdao 266071, China

Y.-F. Zhu, Dr. X.-D. Guo
Institute for Superconducting and Electronic Materials, University of Wollongong, Wollongong, NSW 2522, Australia

X. Yang
Beijing National Laboratory for Condensed Matter Physics, Institute of Physics, Chinese Academy of Sciences (CAS), Beijing 100190, China

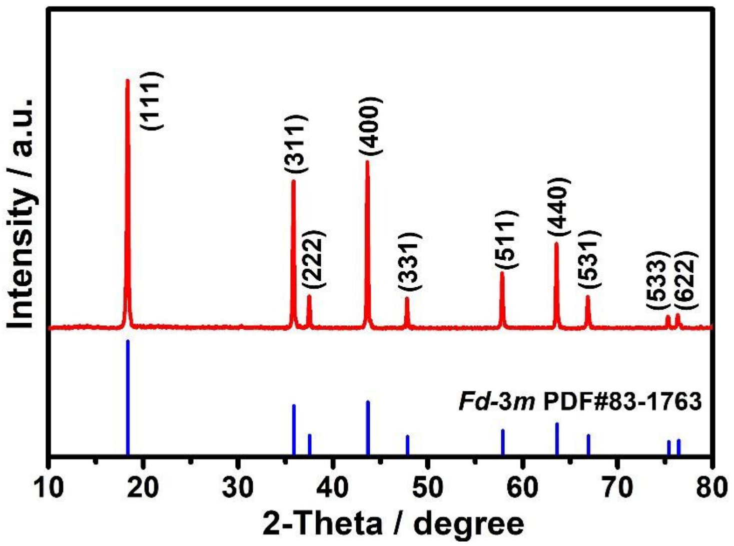

Figure S1. Powder XRD pattern of LMO-CS cathode material.

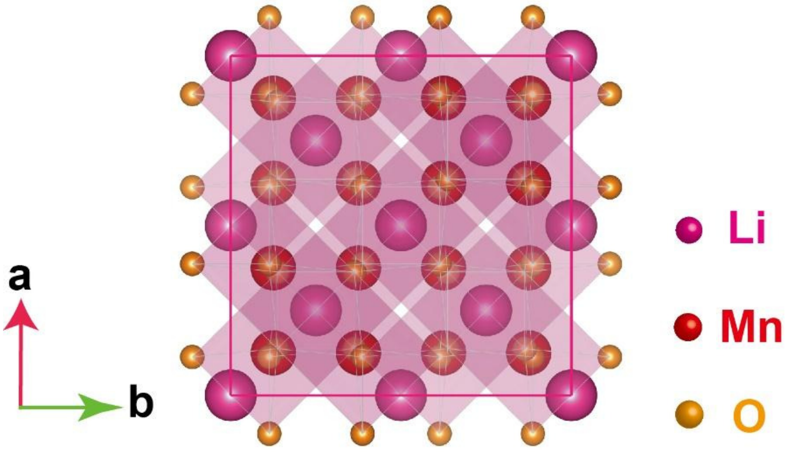

Figure S2. Fd-3m crystal structure of $LiMn_2O_4$ cathode material viewed along the [001] crystallographic direction.

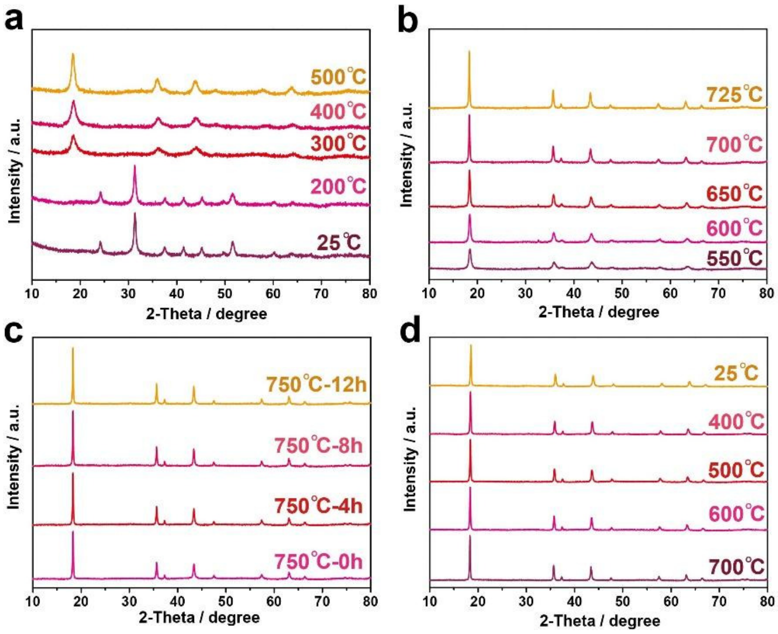

Figure S3. In-situ XRD patterns at different temperatures of precursors concerning LMO-HF cathode material. a, b) XRD patterns from 25 °C to 725 °C during the heating process. c) XRD patterns at 750 °C during the heat preservation process. d) XRD patterns from 700 °C to 25 °C during the cooling process.

Characteristic peaks of $MnCO_3$ from 25 °C to 200 °C matched with the previous report. When the temperature is raised to 300 °C, (111) characteristic peak of LMO-HF cathode material begins to form. When the temperature continues to increase, other main characteristic peaks also progressively appear and the (111) peak becomes much sharper. Meanwhile, with the rise in temperature, all the peaks continuously shift to a lower angle because of thermal expansion and then the peaks experience an opposite evolution after cooling to room temperature.

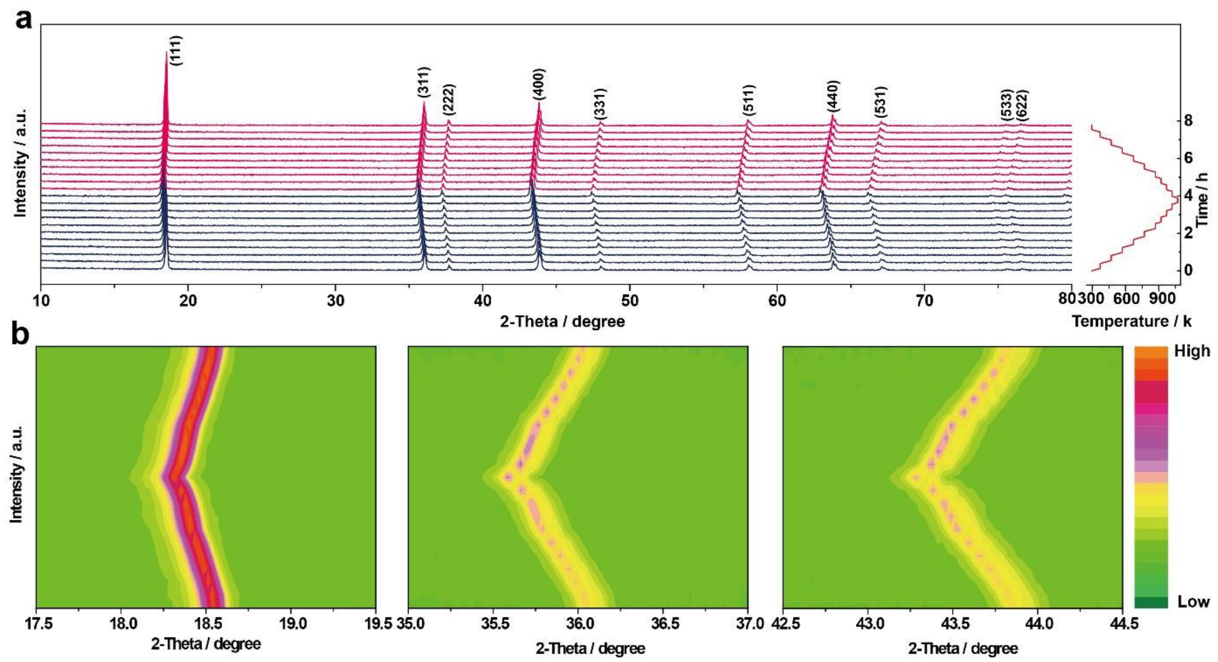

Figure S4. a, b) In-situ XRD patterns at different temperatures of LMO-HF cathode material and intensity contour maps (bird's eye view) concerning the evolution of the main characteristic diffraction peaks.

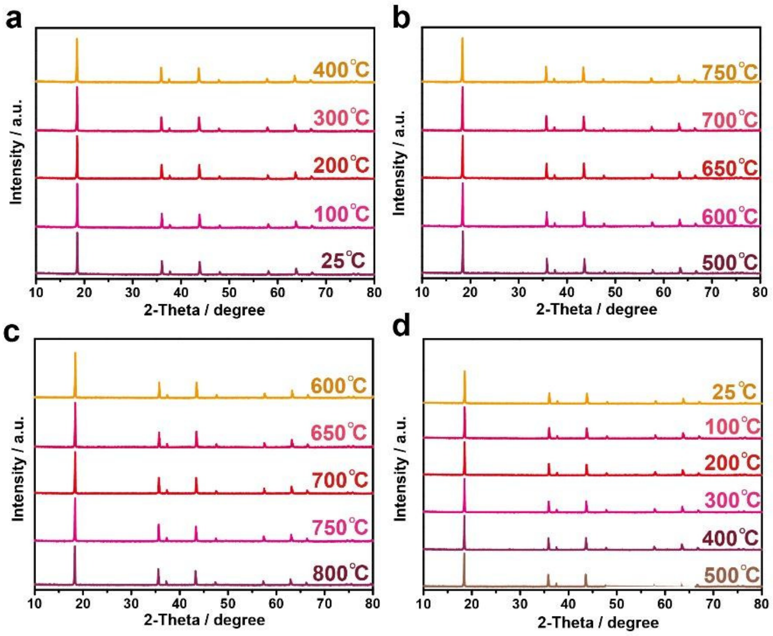

Figure S5. In-situ XRD patterns at different temperatures of LMO-HF cathode material. a, b) XRD patterns from 25 °C to 750 °C during the heating process. c, d) XRD patterns from 800 °C to 25 °C during the cooling process.

Some important information could be obtained from the in-situ XRD at different temperatures. Firstly, the $I_{(111)}/I_{(311)}$ ratio values of LMO-HF cathode material are about 1.84 times higher than that of LMO-CS cathode material during the whole process of heating and cooling, which further confirm the preferentially exposed {111} facets. Secondly, it is worth noting that LMO-HF cathode material still displays excellent structure stability even if the highest heating temperature (1073.15K) surpasses the synthesis temperature (1023.15K). Thirdly, all the peaks of LMO-HF cathode material continuously shift to a lower angle and then the peaks experience an exact opposite evolution after cooling to room temperature, indicating the excellent reversible thermostability of LMO-HF cathode material.

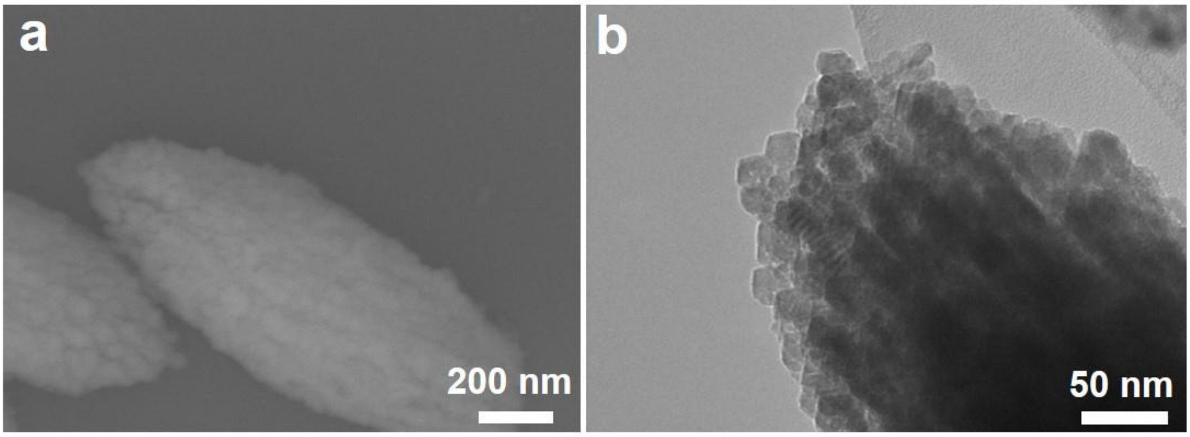

Figure S6. a, b) SEM and TEM images of fusiform $MnCO_3$ precursor.

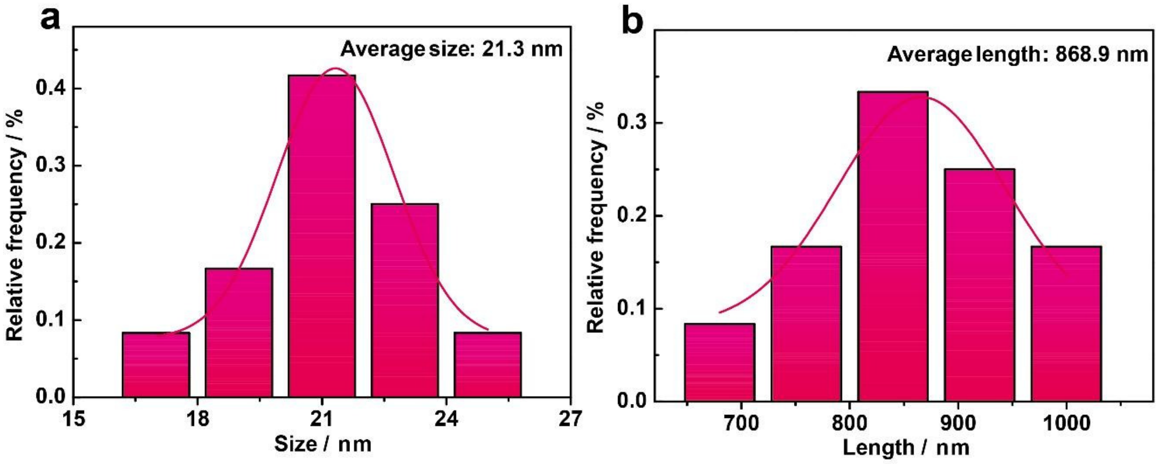

Figure S7. a, b) Size distribution of primary particle and length distribution of second particle concerning fusiform $MnCO_3$ precursor.

The length and diameter distributions were estimated by the software of SMileView. The corresponding results are summarized through mathematical statistics and Gaussian Fitting of the Origin software.

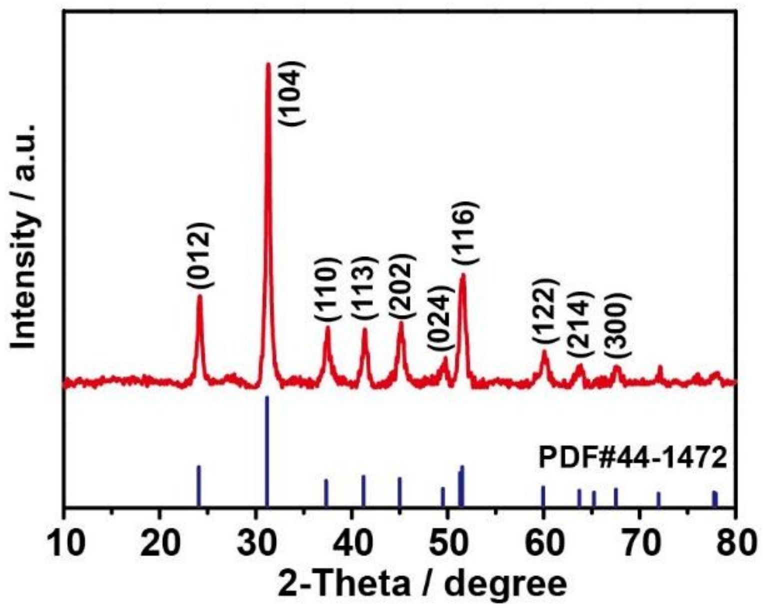

Figure S8. Powder XRD pattern of fusiform $MnCO_3$ precursor.

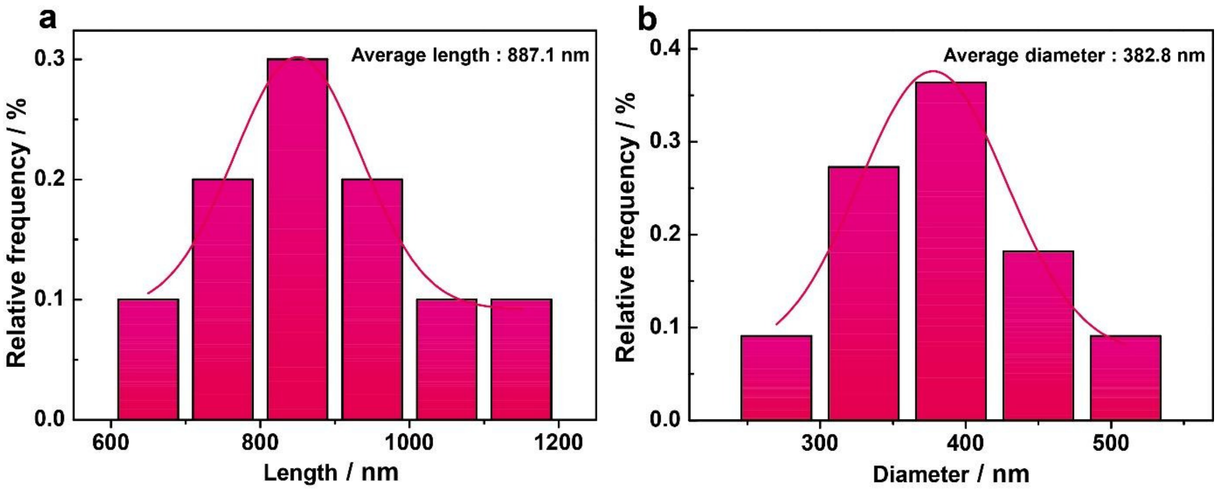

Figure S9. a, b) Length and diameter distribution of second particle concerning LMO-HF cathode material.

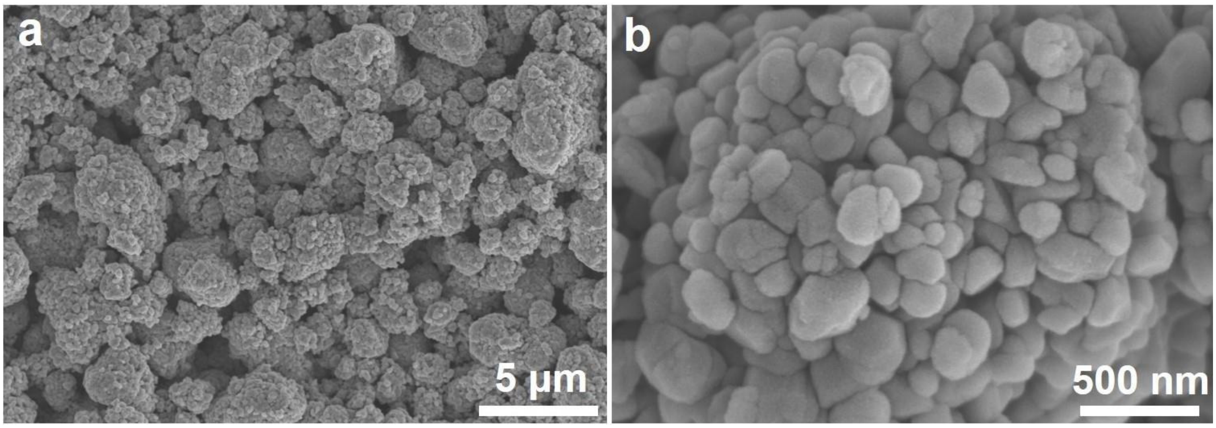

Figure S10. a, b) SEM images of LMO-CS cathode material at different magnifications.

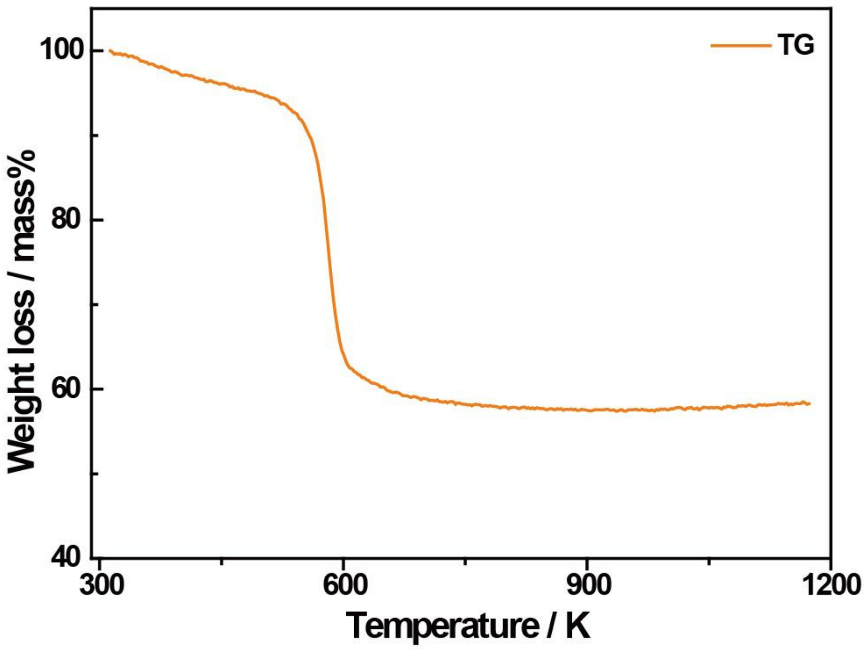

Figure S11. TG curve of precursor concerning LMO-HF cathode material.

TG curve is applied to verify the decomposition process of LMO-HF cathode material. The first weight loss of 6.2% from room temperature to 250 °C in the TG curve can be attributed to the removal of hydrate water. The second significant weight loss of 34.8% is assigned to the thermal decomposition of $MnCO_3$ and lithiation reaction process. Finally, pure phase of $LiMn_2O_4$ cathode material is formed. It is noted that the experimental weight loss for the formation of $LiMn_2O_4$ is 41.5%, which fits well theoretical value of 38.9%.

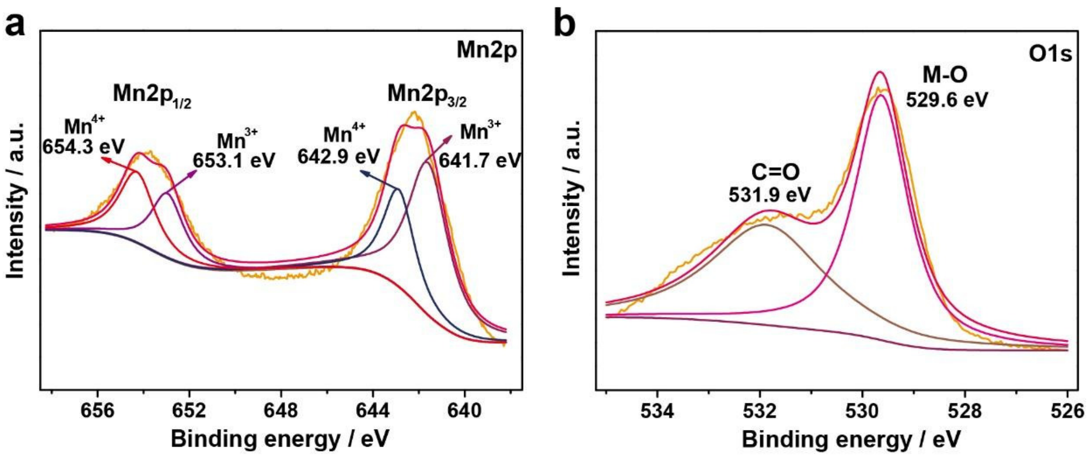

Figure S12. a, b) XPS spectra of LMO-HF cathode material for Mn2p and O1s.

The Mn2p spectrum includes two peaks that can be ascribed to manganese $2p_{3/2}$ and $2p_{1/2}$ with a spin-splitting separation of 11.65 eV. Both the manganese $2p_{3/2}$ and $2p_{1/2}$ regions can be deconvoluted into two peaks, indicating different chemical states of Mn. The peaks at 641.7 and 642.9 eV of the $Mn2p_{3/2}$ spectrum are attributed to Mn^{3+} and Mn^{4+}, respectively, as are those at 653.1 eV for Mn^{3+} and 654.3 eV for Mn^{4+} in the $Mn2p_{1/2}$ spectrum. The O1s XPS spectrum is mainly composed of two peaks, which are assigned to the oxygen in M–O (M refers to the metal ion) existing in the crystal lattice and the organic oxygen, respectively.

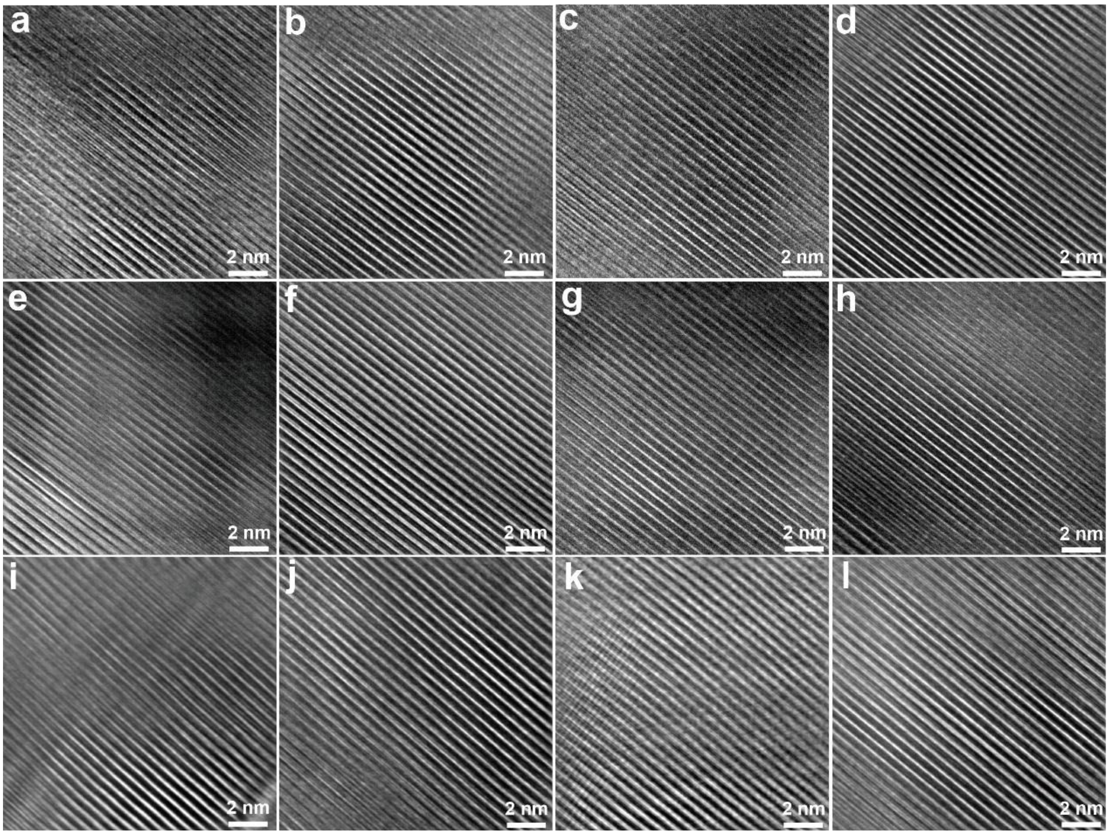

Figure S13. a–l) Filtered HR-TEM images at different sites of LMO-HF cathode material.

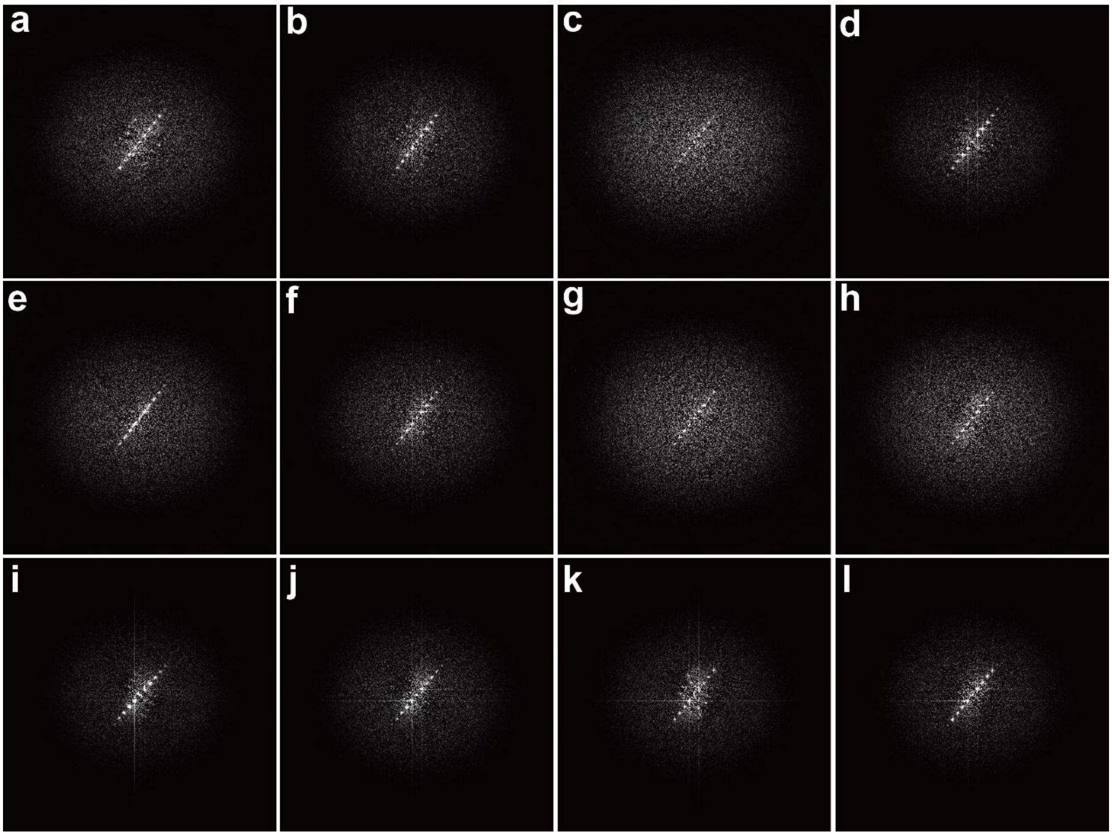

Figure S14. a–l) Filtered FFT images at different sites of LMO-HF cathode material.

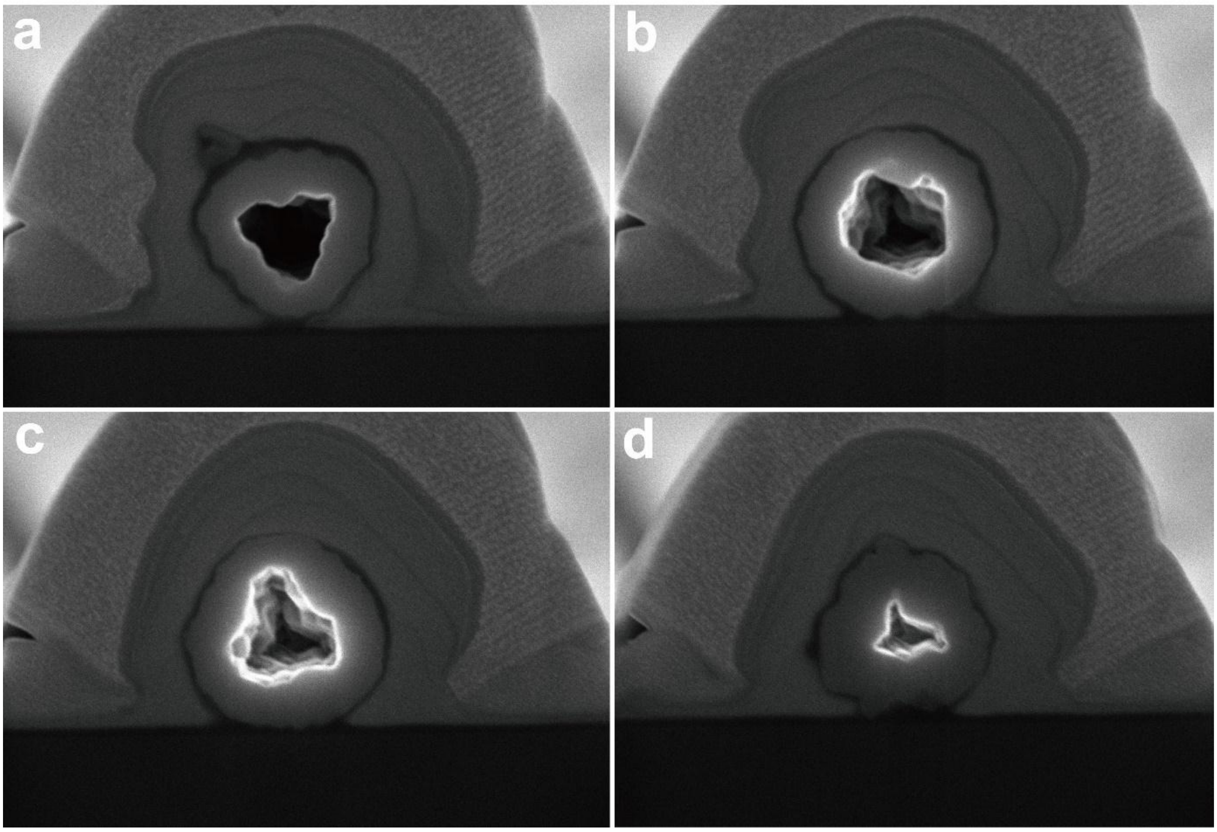

Figure S15. a–d) Original 3D reconstructed cross-sectional images at different state of LMO-HF cathode material.

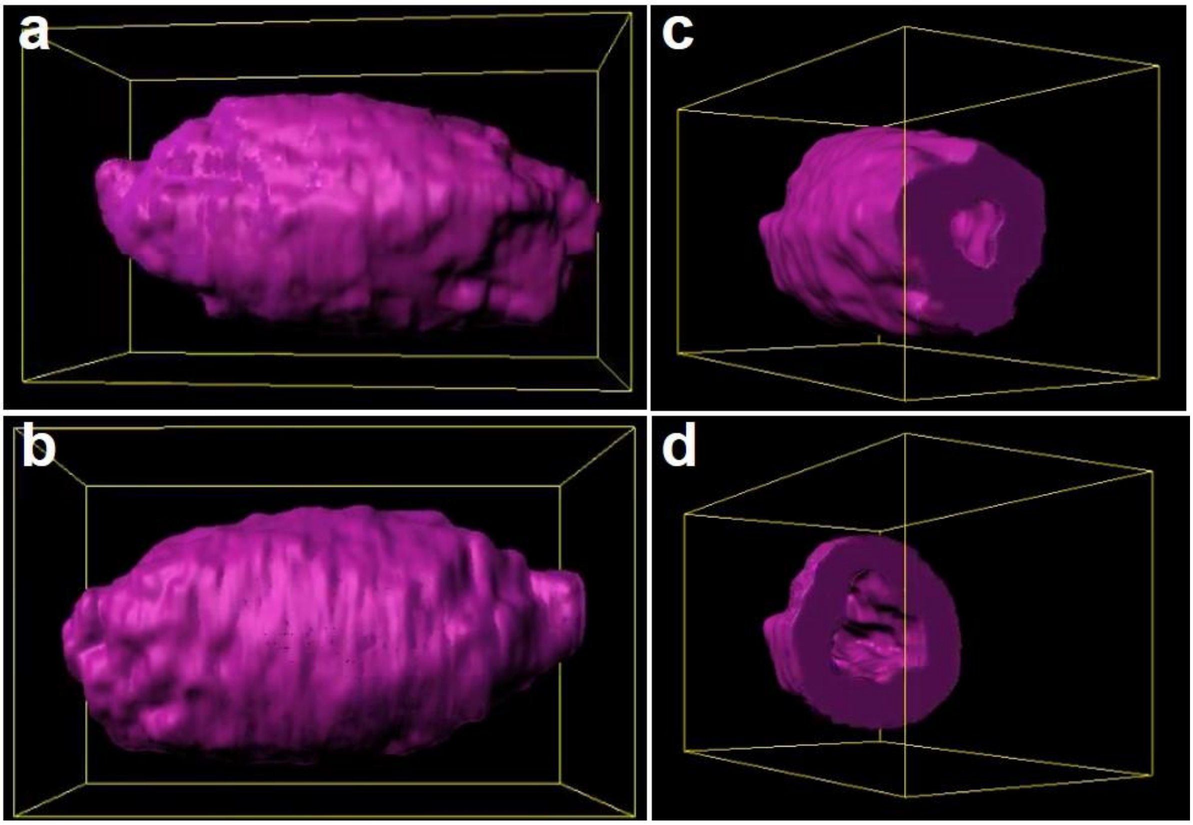

Figure S16. a–d) 3D reconstructed cross-sectional images at different state of LMO-HF cathode material viewed from different angles.

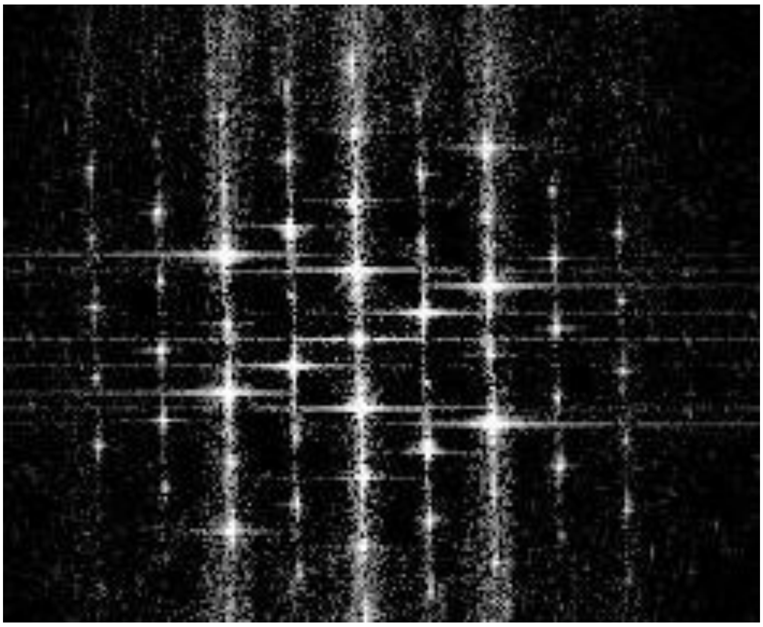

Figure S17. Typical FFT pattern of HAADF-STEM concerning LMO-HF cathode material viewed along the [110] crystallographic direction.

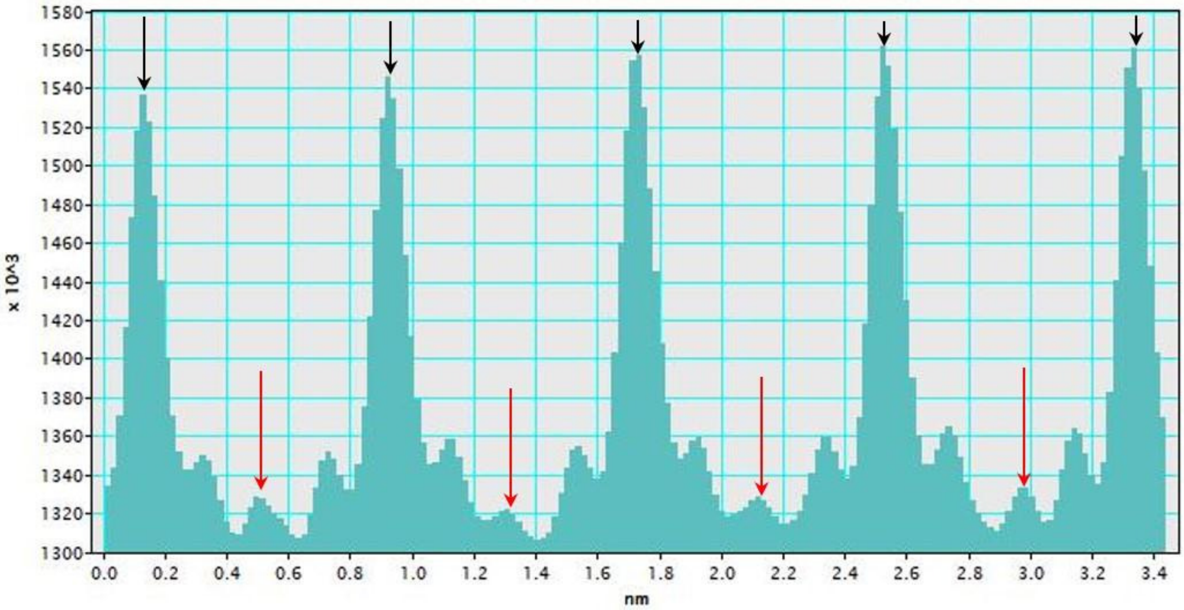

Figure S18. Line profile of HAADF-STEM concerning LMO-HF cathode material with the Mn octahedral sites (black arrows) and the empty octahedral sites (red arrows), respectively.

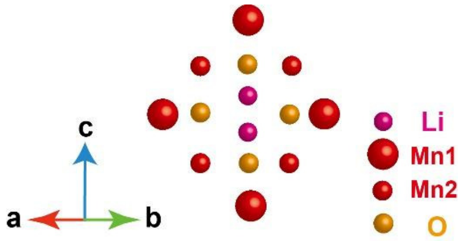

Figure S19. Demonstration of atomic arrangements concerning $LiMn_2O_4$ cathode material viewed along the [110] crystallographic direction.

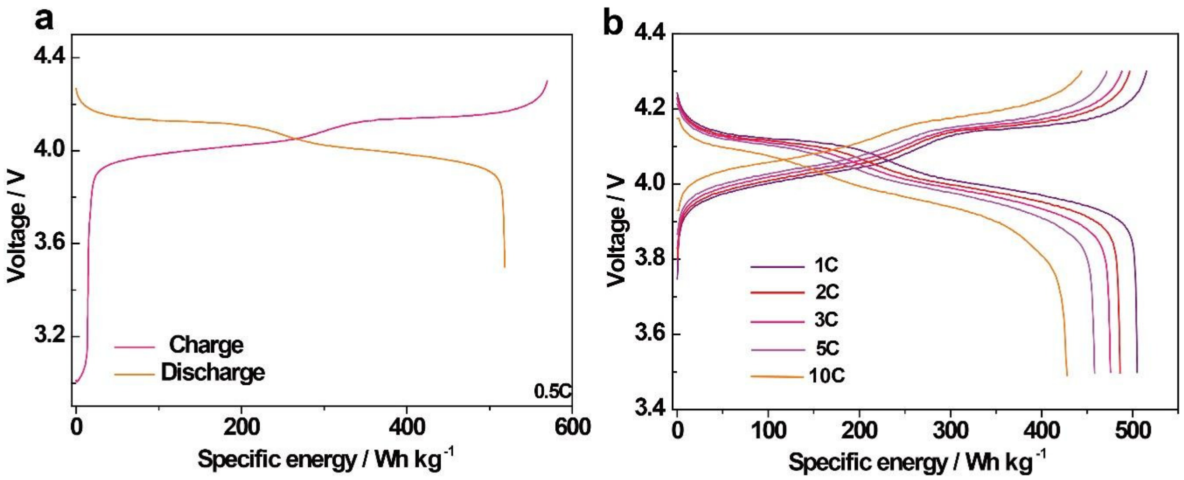

Figure S20. Electrochemical performance of LMO-HF electrode. a) Galvanostatic charge/discharge curves versus specific energy in the first cycle with a current density of 0.5 C at 25 °C. b) Galvanostatic charge/discharge curves versus specific energy at different rate at 25 °C.

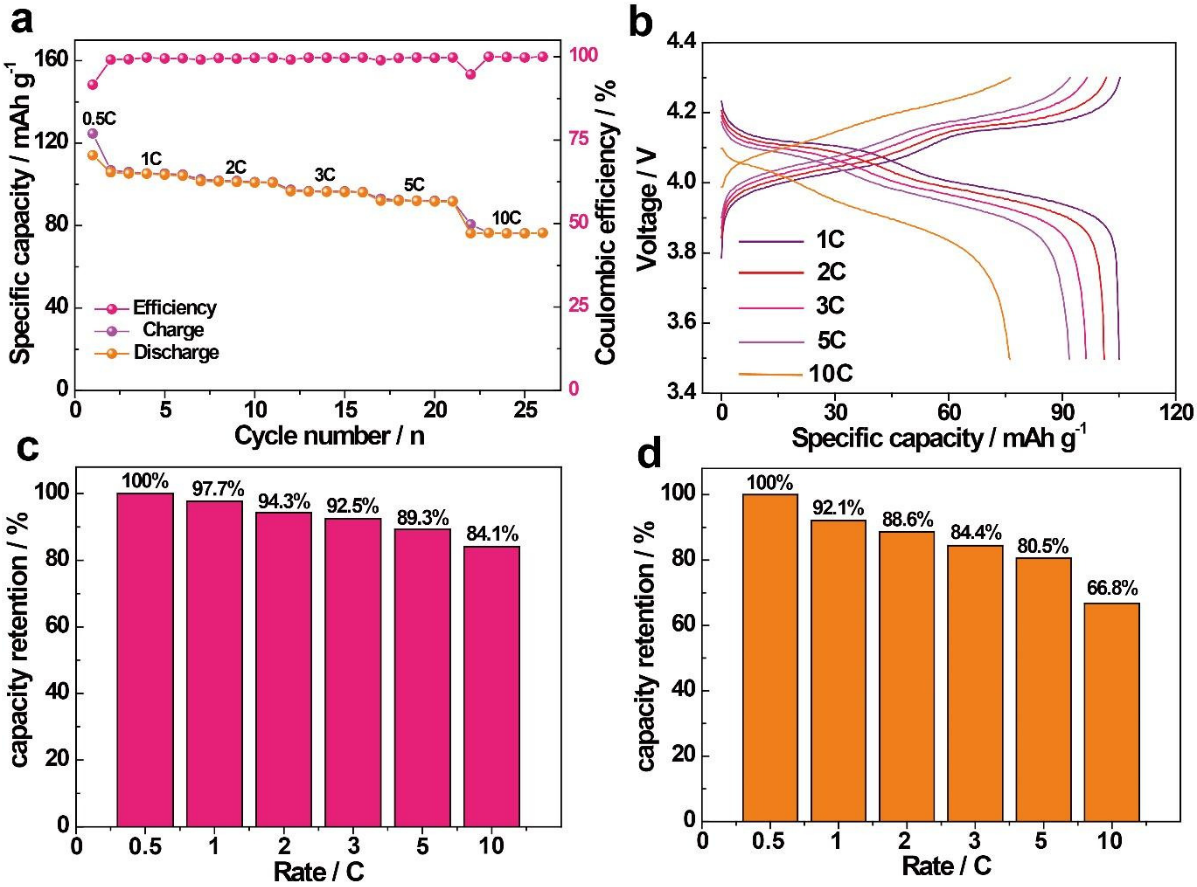

Figure S21. a, b) Rate performance of LMO-CS electrode and corresponding galvanostatic charge/discharge curves versus specific capacity at various rates at 25 °C. c, d) The discharge specific capacity retentions at different rate of LMO-HF and LMO-CS electrodes at 25 °C, respectively.

a

	A	B	C	D
1	Equation	y = a + b*x		
2	Weight	No Weighting		
3	Residual Sum of Squares	0.03622		
4	Pearson's r	0.98869		
5	Adj. R-Square	0.96627		
6			Value	Standard Error
7	B	Intercept	-0.21924	0.18949
8		Slope	88.30472	9.47017

b

	A	B	C	D
1	Equation	y = a + b*x		
2	Weight	No Weighting		
3	Residual Sum of Squares	0.03038		
4	Pearson's r	0.99398		
5	Adj. R-Square	0.98198		
6			Value	Standard Error
7	C	Intercept	-0.30937	0.17353
8		Slope	111.2336	8.67246

c

	A	B	C	D
1	Equation	y = a + b*x		
2	Weight	No Weighting		
3	Residual Sum of Squares	0.02108		
4	Pearson's r	-0.99213		
5	Adj. R-Square	0.97649		
6			Value	Standard Error
7	D	Intercept	0.19962	0.14455
8		Slope	-80.9607	7.22416

d

	A	B	C	D
1	Equation	y = a + b*x		
2	Weight	No Weighting		
3	Residual Sum of Squares	0.03184		
4	Pearson's r	-0.9869		
5	Adj. R-Square	0.96096		
6			Value	Standard Error
7	E	Intercept	0.01135	0.17766
8		Slope	-76.81643	8.87904

e

	A	B	C	D
1	Equation	y = a + b*x		
2	Weight	No Weighting		
3	Residual Sum of Squares	0.0066		
4	Pearson's r	0.98952		
5	Adj. R-Square	0.9583		
6			Value	Standard Error
7	B	Intercept	-0.22144	0.14879
8		Slope	62.37496	9.10187

f

	A	B	C	D
1	Equation	y = a + b*x		
2	Weight	No Weighting		
3	Residual Sum of Squares	0.01141		
4	Pearson's r	0.98805		
5	Adj. R-Square	0.95248		
6			Value	Standard Error
7	C	Intercept	-0.26584	0.19558
8		Slope	76.68483	11.96379

g

	A	B	C	D
1	Equation	y = a + b*x		
2	Weight	No Weighting		
3	Residual Sum of Squares	0.00761		
4	Pearson's r	-0.98882		
5	Adj. R-Square	0.95553		
6			Value	Standard Error
7	D	Intercept	0.25239	0.15978
8		Slope	-64.81055	9.77396

h

	A	B	C	D
1	Equation	y = a + b*x		
2	Weight	No Weighting		
3	Residual Sum of Squares	0.00653		
4	Pearson's r	-0.98681		
5	Adj. R-Square	0.94757		
6			Value	Standard Error
7	E	Intercept	0.09492	0.14794
8		Slope	-55.15751	9.04992

Figure S22. a–d) The results of linear fitting concerning peak current versus square root of the scan rate at different oxidation and reduction peaks for LMO-HF electrode. e–h) The results of linear fitting concerning peak current versus square root of the scan rate at different oxidation and reduction peaks for LMO-CS electrode.

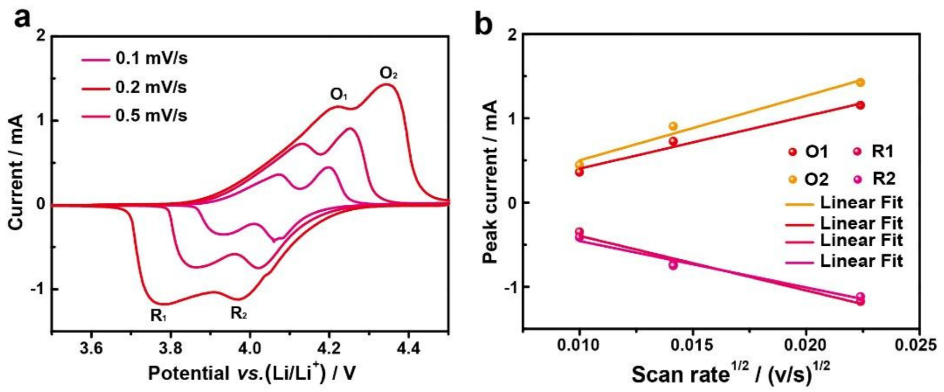

Figure S23. a, b) Cyclic voltammograms at different scan rates and the plotting of peak current versus square root of the scan rate at different oxidation and reduction peaks for LMO-CS electrode.

In order to estimate Li^+ apparent diffusion coefficient of the LMO-HF and LMO-CS electrodes, the CV experiment at various sweeping rates was carried out and the corresponding result was calculated from the slope of peak current versus the square root of the scan rate ($v^{1/2}$) according to the Randles-Sevcik Equation:

$$I_p = 0.4463n^{3/2}F^{3/2}CSR^{-1/2}T^{-1/2}D_{cv}{}^{1/2}v^{1/2}$$

where I_p (A) is peak current, n is the number of electrons per reaction species, F is Faraday constant (96485 C mol^{-1}), C (mol cm^{-3}) is the bulk concentration in moles per cubic centimeter (0.02378 mol cm^{-3}), S (cm^2) is the area of the electrode, R is the gas constant (8.314 J mol^{-1} K^{-1}), T (K) is the absolute temperature, D_{CV} (cm^2 s^{-1}) is the Li^+ apparent diffusion coefficient, and v (V s^{-1}) is potential scan rate in volts per second. Accordingly, the evaluated diffusion coefficients at different peaks of the LMO-HF and LMO-CS electrodes are presented in TableS2 and 3, respectively .

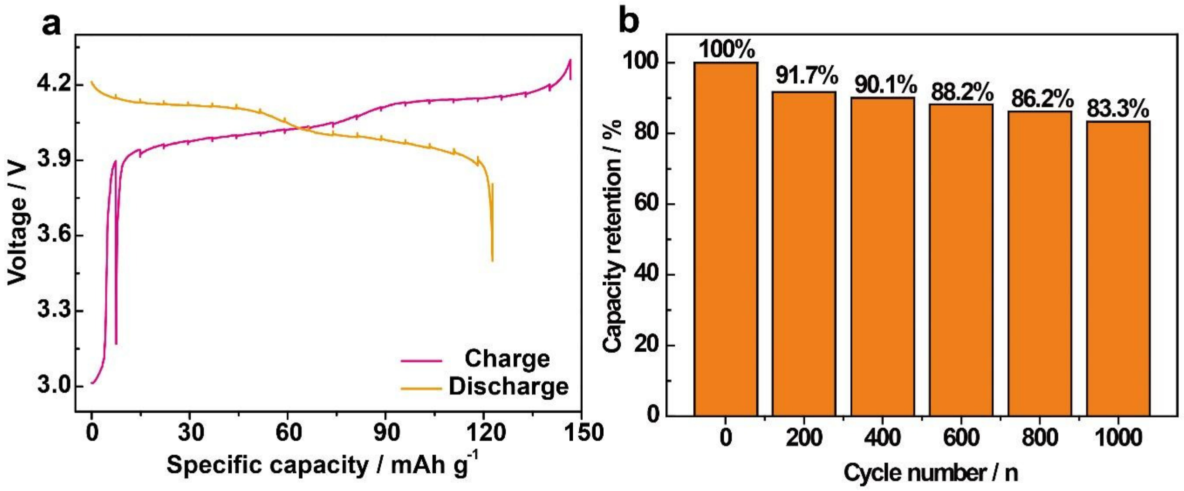

Figure S24. Electrochemical performance of LMO-HF electrode. a) GITT curves of LMO-HF electrode in the first cycle at 25 °C. b) The discharge capacity retentions are selected every 200 cycles with a current density of 1C at 25 °C.

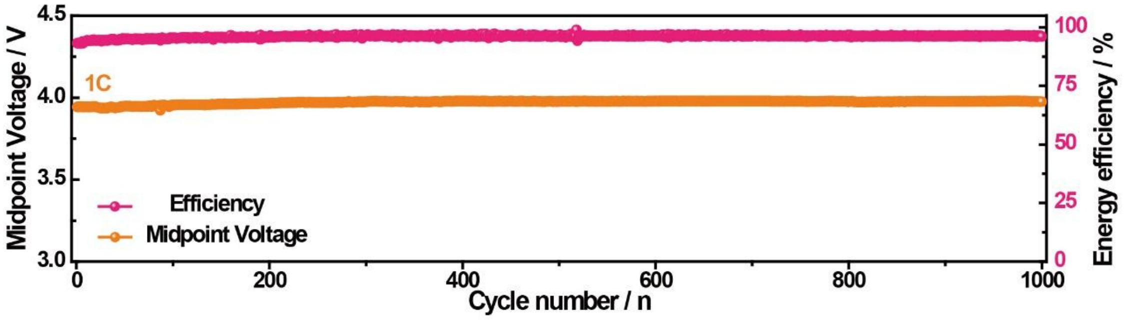

Figure S25. Midpoint voltage and energy efficiency of LMO-HF electrode during 1000 cycles with a current density of 1C at 25 °C.

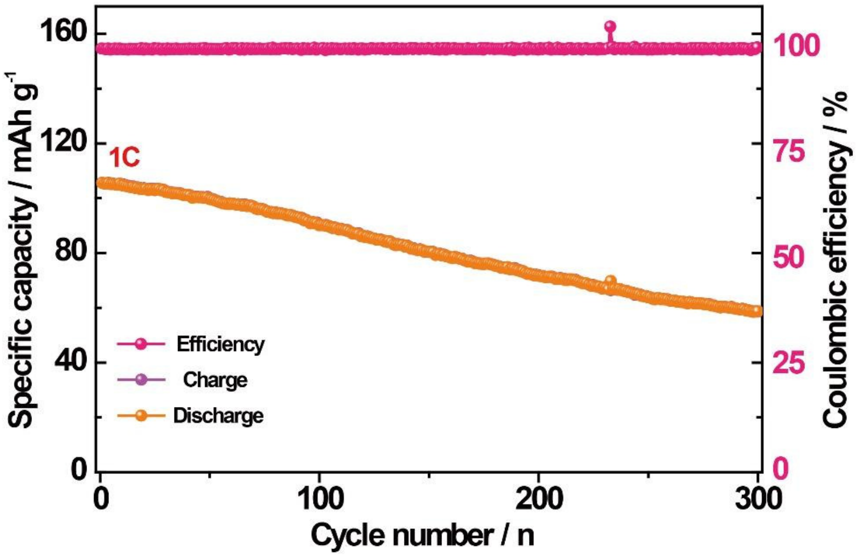

Figure S26. Cycling performance of LMO-CS electrode during 300 cycles with a current density of 1C at 25 °C.

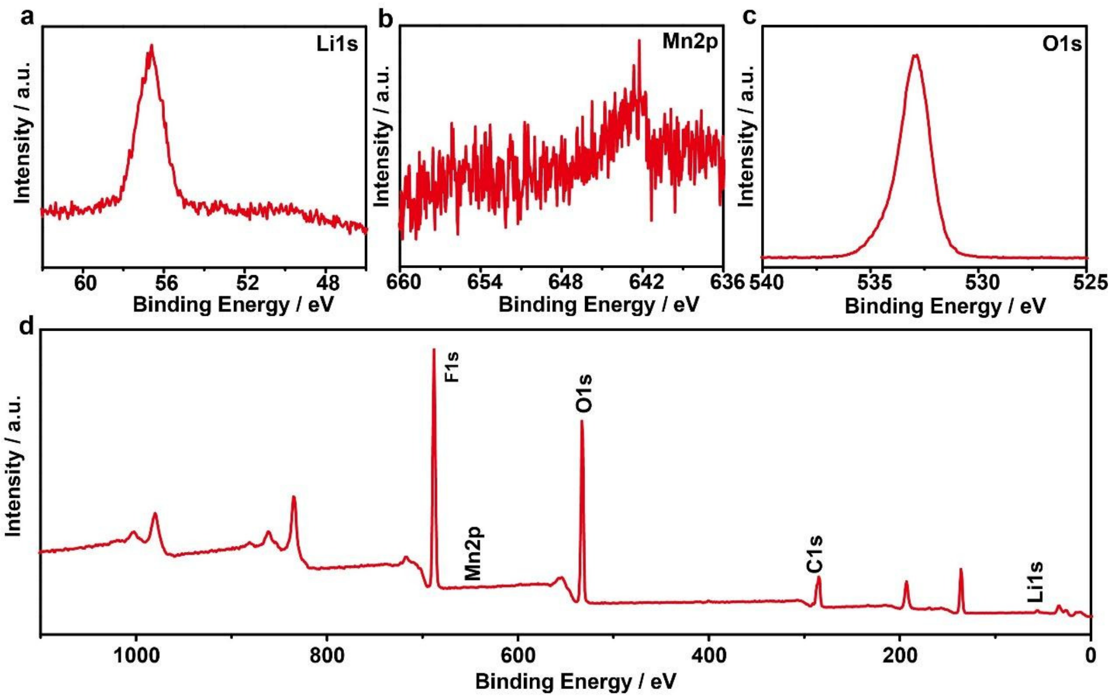

Figure S27. a-d) Ex-situ XPS spectra on the surface of lithium metal anode collected after 1000 cycles with a current density of 1C at 25 °C.

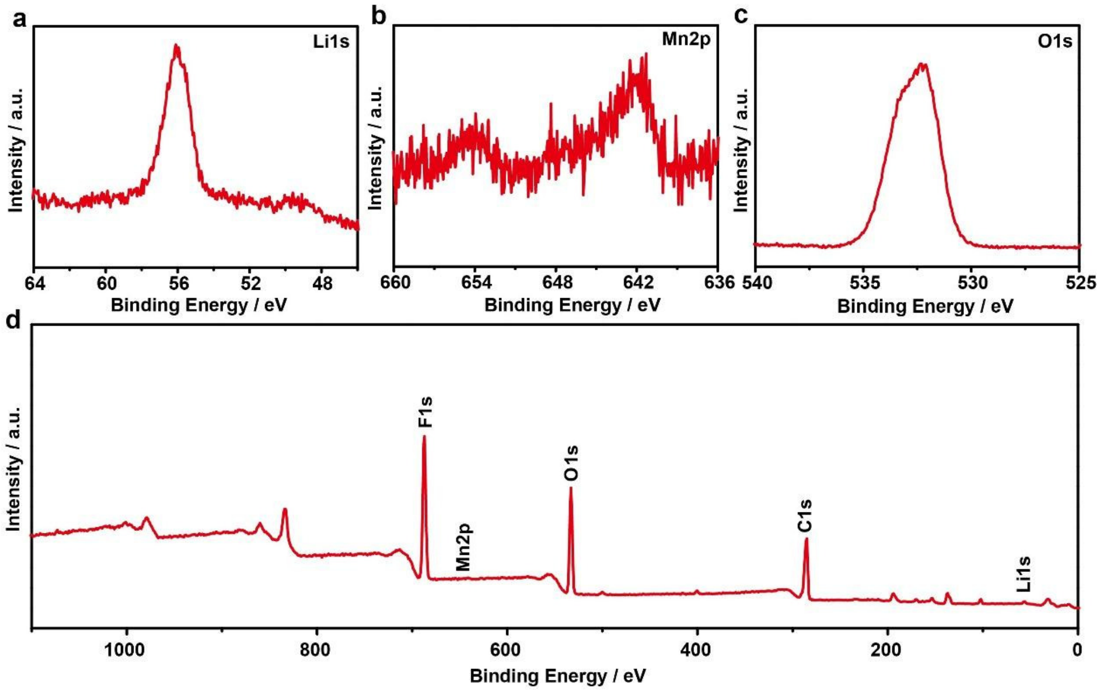

Figure S28. a-d) Ex-situ XPS spectra on the surface of lithium metal anode collected after 500 cycles with a current density of 0.5C at 60 °C.

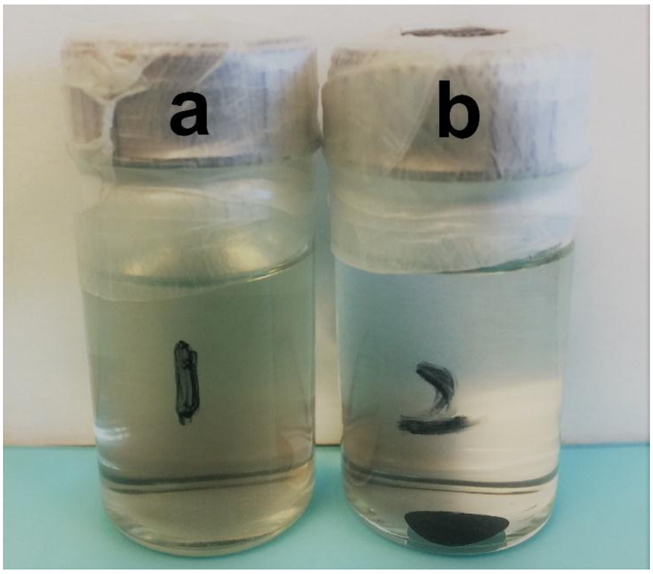

Figure S29. Photographs of the electrolyte stored at 60 °C for 24 h. a) without LMO-HF electrode, b) with LMO-HF electrode.

Table S1. ICP-MS results of the LMO-HF and LMO-CS cathode materials.

**Cathode Materials**	**Measured atomic ratio**	
	**Li**	**Mn**
LMO-HF	0.998	2.001
LMO-CS	0.997	1.998

Table S2. Summary of the CV results obtained at different scanning rates and the Li^+ diffusion coefficients concerning the LMO-HF electrode.

Scanning rate mV / S	Current values / mA				Voltage values / V					
	C(O1)	C(O2)	C(R1)	C(R2)	V(O1)	V(O2)	V(R1)	V(R2)	V(OR1)	V(OR2)
**0.1**	0.568	0.714	0.564	0.685	4.058	4.174	3.958	4.077	0.100	0.097
**0.2**	1.107	1.337	0.958	1.115	4.068	4.177	3.943	4.079	0.125	0.098
**0.5**	1.871	2.282	1.726	1.836	4.091	4.196	3.934	4.071	0.157	0.125
**0.8**	2.185	2.752	2.011	2.066	4.098	4.204	3.925	4.064	0.173	0.140
D_{Li^+} **(10^{-10} cm^2 s^{-1})**	3.098	4.914	2.344	2.607						

C(O): cathodic peak current, C(R): anodic peak current, V(O): cathodic peak voltage, V(R): anodic peak voltage, V(OR): the separation between V(O) and V(R). The number 1 and 2 denote the redox couple at lower and higher potential, respectively.

Table S3. Summary of the CV results obtained at different scanning rates and the Li^+ diffusion coefficients concerning the LMO-CS electrode.

Scanning rate mV / S	Current values / mA				Voltage values / V					
	C(O1)	C(O2)	C(R1)	C(R2)	V(O1)	V(O2)	V(R1)	V(R2)	V(OR1)	V(OR2)
**0.1**	0.359	0.444	0.349	0.414	4.074	4.198	3.926	4.064	0.148	0.134
**0.2**	0.726	0.904	0.734	0.749	4.129	4.252	3.859	4.019	0.270	0.233
**0.5**	1.154	1.423	1.176	1.119	4.218	4.342	3.776	3.969	0.442	0.373
D_{Li^+} **(10^{-10}cm^2 s^{-1})**	1.545	2.337	1.669	1.209						

C(O): cathodic peak current, C(R): anodic peak current, V(O): cathodic peak voltage, V(R): anodic peak voltage, V(OR): the separation between V(O) and V(R). The number 1 and 2 denote the redox couple at lower and higher potential, respectively.

Table S4. Manganese dissolution amount after cycles tested by ICP-MS (based on the mass).

**Electrodes**	**Different Temperature**	**Mn (wt%)**
LMO-HF	After 500 cycles at 0.5C (60℃)	0.745
LMO-HF	After 1000 cycles at 1C (25℃)	0.386
LMO-CS	After 300 cycles at 1C (25℃)	0.923
